# Construction and Analysis of the Dysregulated ceRNA Network and Identification of Risk Long Noncoding RNAs in Breast Cancer

**DOI:** 10.3389/fgene.2021.664393

**Published:** 2021-06-04

**Authors:** Xiaojie Su, Zhaoyan Yu, Yuexin Zhang, Jiaxin Chen, Ling Wei, Liang Sun

**Affiliations:** ^1^College of Medical Laboratory Science and Technology, Harbin Medical University, Daqing, China; ^2^Department of Otorhinolaryngology, Shandong Provincial Hospital Affiliated to Shandong First Medical University and Shandong Academy of Medical Sciences, Jinan, China; ^3^School of Medical Informatics, Harbin Medical University, Daqing, China; ^4^College of Artificial Intelligence and Big Data for Medical Sciences, Shandong First Medical University & Shandong Academy of Medical Sciences, Jinan, China; ^5^Hwa Mei Hospital, University of Chinese Academy of Sciences, Ningbo, China

**Keywords:** breast cancer, risk lncRNA, competitive endogenous RNA, ceRNA network, random walk with restart, prognostic signature

## Abstract

Breast cancer (BRCA) is the second leading cause of cancer-related mortality in women worldwide. However, the molecular mechanism involved in the development of BRCA is not fully understood. In this study, based on the miRNA-mediated long non-coding RNA (lncRNA)–protein coding gene (PCG) relationship and lncRNA–PCG co-expression information, we constructed and analyzed a specific dysregulated lncRNA–PCG co-expression network in BRCA. Then, we performed the random walk with restart (RWR) method to prioritize BRCA-related lncRNAs through comparing their RWR score and significance. As a result, we identified 30 risk lncRNAs for BRCA, which can distinguish normal and tumor samples. Moreover, through gene ontology and Kyoto Encyclopedia of Genes and Genomes pathway analysis, we found that these risk lncRNAs mainly synergistically exerted functions related to cell cycle and DNA separation and replication. At last, we developed a four-lncRNA prognostic signature (including AP000851.1, LINC01977, MAFG-DT, SIAH2-AS1) and assessed the survival accuracy of the signature by performing time-dependent receiver operating characteristic (ROC) analysis. The areas under the ROC curve for 1, 3, 5, and 10 years of survival prediction were 0.68, 0.61, 0.62, and 0.63, respectively. The multivariable Cox regression results verified that the four-lncRNA signature could be used as an independent prognostic biomarker in BRCA. In summary, these results have important reference value for the study of diagnosis, treatment, and prognosis evaluation of BRCA.

## Introduction

Breast cancer (BRCA) is one of the most prevalent malignancies and is the second leading cause of cancer-related mortality in women worldwide (Bray et al., 2018; DeSantis et al., 2019). Although there have been several advancements in both surgical and adjuvant therapy, BRCA remains a significant threat to female health due to high incidence and poor prognosis. There is an urgent need for novel and effective biomarkers for clarifying mechanism of early BRCA and providing therapeutic targets for BRCA patients (Chi et al., 2019).

Long non-coding RNAs (lncRNAs) are a group of RNAs with length >200 bp, which serve as key regulators in diverse cellular functions such as development, differentiation, and apoptosis (Ulitsky and Bartel, 2013; Quinn and Chang, 2016; Trovero et al., 2020). The important function of lncRNA is that it can act as a competitive endogenous RNA (ceRNA) to regulate the expression level of other transcripts especially for protein coding gene (PCG) by sponging miRNA (Quinn and Chang, 2016). Growing evidences demonstrated that lncRNAs had been indicated as important molecules in tumorigenesis (Gibb et al., 2011). Herrera-Solorio et al. (2020) showed that lncRNA SOX2-OT modulates an orchestrated resistance mechanism, promoting poor prognosis and human lung malignancy through genetic, epigenetic, and posttranslational mechanisms. Xiao et al. (2020) found that the lncRNA MAFG-AS1, which is highly expressed in bladder urothelial carcinoma, is correlated with aggressive characteristics and poor prognosis of bladder urothelial carcinoma. As for BRCA, IRNAS HOTAIR, SPRY4-IT1, GAS5, MATAR25, PANDAR, and MATAR25, new players in tumor development and prognosis, have shown owning potential clinical applications in BRCA (Soudyab et al., 2016; Nagini, 2017; Chang et al., 2020). In addition, viable ways have been considered to predict the potential BRCA lncRNAs by performing high-throughput data based on bioinformatics methods (Guo et al., 2018; Chi et al., 2019; Wang et al., 2019).

In our study, by analyzing the BRCA expression profile from The Cancer Genome Atlas (TCGA) and lncRNA-related databases, we constructed and analyzed a specific dysregulated BRCA-associated lncRNA-PCR ceRNA network and performed the random walk with restart (RWR) method to prioritize BRCA-related lncRNAs through comparing their RWR score and significance. At last, we identified 30 risk lncRNAs associated with BRCA and constructed a prognostic signature based on the TCGA expression data with clinical survival characters. This study has important reference value to accelerate the discovery of molecular biomarkers for the study of diagnosis, treatment, and prognosis evaluation of BRCA.

## Materials and Methods

### Datasets Across Breast Cancer

The PCG and lncRNA expression profiles of BRCA with FPKM values were obtained from the UCSC Cancer Browser^1^, which provided an open-access portal to download data from TCGA. In total, we acquired 1,222 BRCA samples that were involved in 1,075 patients with complete clinical follow-up information. We performed a two-step filter for PCG and lncRNA expression profiles; the aim is to ensure detection reliability and reduce noise. First, we extracted only paired patient samples with tumor and adjacent nontumor tissue for differentially analysis. Second, lncRNAs or PCGs with an average expression value of less than 1 were removed in the tumor and adjacent nontumor tissue. Finally, we obtained 224 tumor and adjacent nontumor tissue from 112 patients, including 1,251 lncRNAs and 13,356 PCGs. Processed gene expression data and clinical data were provided in Supplementary Material. For the calculation of Pearson correlation coefficient (PCC), log2 transformation was performed to lncRNA/TF expression profiles with raw expression values.

### Breast Cancer-Associated Known PCGs

We downloaded BRCA-related PCGs from DisGeNET (v7.0) (Piñero et al., 2020). DisGeNET is a discovery platform containing one of the largest publicly available collections of genes associated with human diseases, which integrates data from expert curated repositories and the scientific literature (Piñero et al., 2020). We extracted 318 PCGs associated with BRCA from DisGeNET (Supplementary Table 1).

### Identification of the Differentially Expressed PCGs/LncRNAs

Fold change and statistical significance were computed for each PCG/lncRNA in expression profiles by limma package, which is a common, effective R/Bioconductor software package for differential expression analyses (Ritchie et al., 2015). The lncRNAs and PCGs with *P* < 0.05 or | log2 fold change| > 1 were considered to be differentially expressed (DE) lncRNAs/PCGs.

### Construction of a Specific LncRNA–PCG ceRNA Network for Breast Cancer

We performed two steps to construct specific LncRNAs-PCGs ceRNA network for breast cancer (SLGCeNBC). Firstly, we calculated the correlation of co-expression between DE PCGs and lncRNAs using the PCC method. PCC can be used to measure the linear relationship between lncRNA and PCG expression (Zhang et al., 2018). In this study, we considered that the lncRNA–PCG pairs with PCC > 0.5 and *P* < 0.01 showed a potential expression correlation. All lncRNA–PCG pairs meeting the threshold were merged into the lncRNA–PCG co-expression network.

Secondly, we performed the hypergeometric test to identify miRNA-mediated lncRNA–PCG pairs. Previous studies proposed and demonstrated that the number of common targeting miRNAs between lncRNAs and PCGs determined the ceRNA cross-talk strength. More common targeting miRNAs could produce a more strength ceRNA cross-talk pair. Thus, we downloaded the interaction of miRNA–PCG/lncRNA from StarBase (Li et al., 2014), mirTarbase (Chou et al., 2018), TargetScan (Grimson et al., 2007), LncBase (Karagkouni et al., 2020), and MiRcode (Jeggari et al., 2012) databases. We downloaded 714,288 miRNA–PCG interaction pairs and 766,809 miRNA–lncRNA interaction pairs, including 13,295 PCGs, 13257 lncRNAs, and 2,593 miRNAs. Then, we used these data to perform the hypergeometric test (Feng et al., 2019). We considered *P*-value < 0.01 as statistically significant. The *P*-value was measured as the following:

$$p=1-\sum_{i=0}^{r-1}\frac{\left(\begin{array}{c} t\\ i \end{array}\right)\,\left(\begin{array}{c} m-t\\ n-i \end{array}\right)}{\left(\begin{array}{c} m\\ n \end{array}\right)}$$

where *m* stands for the total number of human genome miRNAs, *t* stands for the number of miRNAs interacting with the PCG, *n* stands for the number of miRNAs interacting with the lncRNAs, and *r* stands for the number of miRNAs shared between PCGs and lncRNAs.

### Random Walking Analysis

Here, we performed RWR to determine ranking for BRCA-related lncRNAs. A random walk in network was defined as an iterative walker’s transition from its certain node to a randomly selected neighbor that started from a given node (e.g., “PCG *x*” was a known PCG associated with BRCA) (Zhang et al., 2018). The random walk performed had capacity of restart with probability *r* in every time step at node “PCG *x*.” The random walk with restart was defined as the following:

$$p_{t+1=}\,\left(1-r\right)\,W\,p\,t+r\,p\,0$$

where *W* represents the column-normalized adjacency matrix of the network, *p*_*t*_ is a vector whose size is equivalent to the number of nodes in the network, and the *i*-th element holds the probability of being at node *i* at time step *t*.

The initial probability vector *p*_0_ was constructed such that 1 was assigned to the nodes representing known PCGs associated with BRCA, and other nodes with 0. We considered that the role of PCGs related to disease was equivalent in the network. Vector *p* would be in the steady state at time step *t*, where *t* approached infinity as a limit. The iteration would be finished till the change between *p*_*t*_ and *p_*t*__+__1_* falls below 10^–10^.

We scored for each lncRNA to prioritize lncRNAs related to BRCA by performing the RWR algorithm in a specific LncRNA–PCG ceRNA network for breast cancer (SLGCeNBC) and performed statistical significance analysis for the score of every lncRNA. The statistical significance was determined by comparing the scores of lncRNAs in the network following *n* iterations with SLGCeNBC perturbation. To maintain the network topological properties, random sampling without replacement was performed when doing the random disturbance. In iterations, the times that the score of every lncRNA was higher than the real one were recorded as *m*. The *P*-value for every lncRNA was the ratio of *m* and *n*. In this study, *n* was set at 10,000 times.

### Constructing the Prognostic lncRNA Signature

To identify the best score cutoff of the selected lncRNAs for grouping patients most significantly, we employed for optimal cutoff identification by using the R package “maxstat” (Hothorn and Zeileis, 2008). The survival outcomes of the two groups were estimated by Kaplan–Meier analysis (Bland and Altman, 1998). Then, we used the survival-related lncRNAs (log-rank test *P* < 0.05) above to perform Cox regression analysis (Cox, 1972) and construct risk models as follows:

$$\mathrm{Risk}\,\mathrm{Score}=\sum_{i=1}^{n}\left(x_{i}\,e_{i}\right)$$

where *n* is the number of lncRNAs, *e*_*i*_ is the expression value of the lncRNA_*i*_, and *x*_*i*_ is the coefficient of lncRNAs in Cox regression analysis. Finally, we used the signature with the minimum log-rank *P*-value as the best prognostic marker (Guo et al., 2016). Cox regression analysis was performed to explore the predictive independence of the lncRNA signature. R software^2^ with R packages including timeROC, survival was used for statistical analysis, where a *P*-value of < 0.05 was considered statistically significant.

## Results

### Construction of a Specific LncRNA–PCG ceRNA Network for Breast Cancer

Based on the interaction of miRNAs–PCGs/lncRNAs from the public database, we merged all miRNA–PCG pairs and miRNA–lncRNA pairs and then obtained a global PCG–miRNA–lncRNA triple network. PCG and lncRNA which shared at least one common miRNA were reserved. Then, we performed the hypergeometric test to identify PCG–lncRNA pairs with *P*-value < 0.01, which yielded more than candidate 800,000 lncRNA–PCG interactions. All the pairs were merged into a miRNA-mediated lncRNA–PCG network.

Second, based on the expression profile of 112 pairs of tumor and adjacent nontumor tissue, we obtained DE lncRNAs/PCGs of BRCA by limma with *P* < 0.05 and | log2 fold change| > 1, which produced 316 and 2,463 DE lncRNAs and PCGs, respectively. Then, we calculated the Pearson correlation between DE PCGs and lncRNAs with PCC > 0.5 and *P* < 0.01. All the co-expressed lncRNA–PCG pairs were merged into a co-expression network. As for the lncRNA–PCG co-expression network, it contained 307 lncRNAs, 2,105 PCGs, and 57,216 co-expression relationships. Obviously, it is specific for BRCA.

**FIGURE 1:**
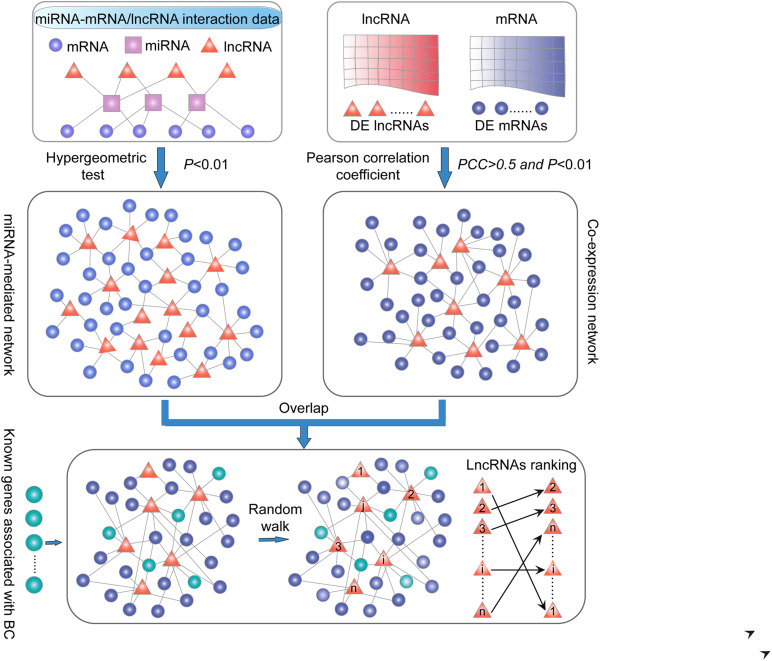
Schematic overview in this study. (i) LncRNA–PCG ceRNA network. PCG and lncRNA shared at least one common miRNA and considered that a *P*-value < 0.01 was significant by applying the hypergeometric test. (ii) LncRNA–PCG co-expression network. The correlation of co-expression between DE PCGs and lncRNAs using the PCC (PCC > 0.5 and *P* < 0.01). (iii) Extracted common lncRNA–PCG pairs in (i) and (ii) to structure SLGCeNBC. (iv) The BRCA-associated known PCGs (seed nodes) were mapped into the SLGCeNBC, and the RWR method was performed on this network. Finally, we the ranked candidate lncRNAs according to the steady probability of RWR.

**FIGURE 2:**
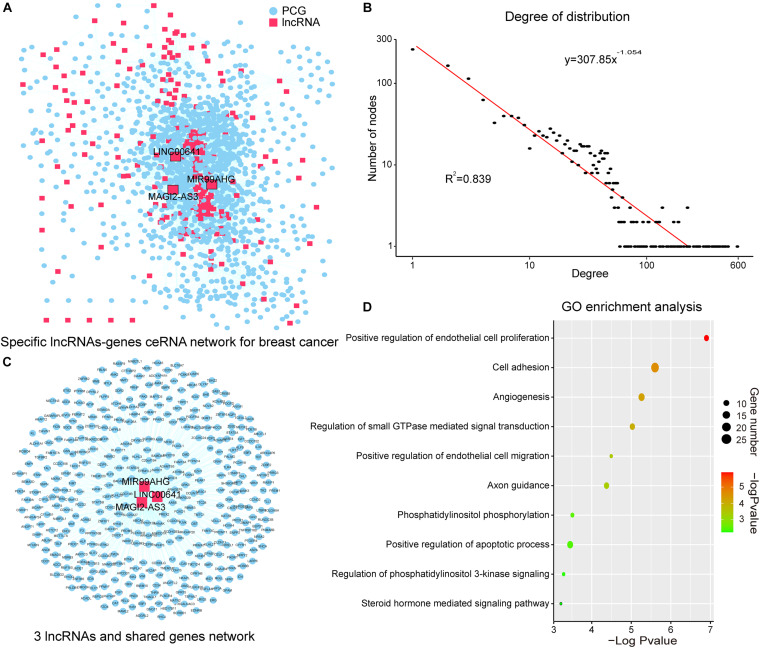
SLGCeNBC and three lncRNAs appeared in two dimensions. **(A)** SLGCeNBC. The red and blue nodes represent lncRNAs and PCGs, respectively. A lncRNA and PCG were connected by an edge if they had the ceRNA and co-expression relationships (Supplementary Table 2). **(B)** The true node degree distribution of SLGCeNBC; the degree distribution of all nodes followed the power law distribution approximately with a slope of –1.054 and *R*^2^ = 0.839. **(C)** The lncRNAs (MAGI2-AS3, MIR99AHG, and LINC00641) are ranked in the top 20 in at least two dimensions (degree, betweenness, and closeness). **(D)** GO enrichment analysis for lncRNAs MAGI2-AS3, MIR99AHG, and LINC00641 through their related PCGs that share at least two of three lncRNAs in SLGCeNBC. The *X*-axis and bubble color are the –log10 of *P*-value; the *Y*-axis is the names of the GO terms. Bubble size indicates the number of PCGs annotated to the GO term.

Finally, we extracted common lncRNA–PCG pairs in the above two networks to construct SLGCeNBC. We defined the PCGs or lncRNAs as the nodes of the network. The whole identification process is shown in Figure 1. In total, 259 lncRNAs, 1,384 PCGs, and 21,702 edges were included in SLGCeNBC (Figure 2A and Supplementary Table S2).

### Topological Analysis of SLGCeNBC

The degree distribution of all nodes followed the power law distribution approximately with *R*^2^ = 0.839 (Figure 2B). A handful of nodes with a high degree in the networks were defined as hubs that linked many nodes; most nodes in networks had few interactions. We firstly analyzed the topological properties of the SLGCeNBC and calculated the degree, closeness, and betweenness of the SLGCeNBC, respectively. We ranked all the nodes’ topological features of the network and listed the top 20 nodes of degree, betweenness, and closeness, respectively (Table 1). Interestingly, we found that three lncRNAs (MAGI2-AS3, MIR99AHG, and LINC00641) appeared in at least two dimensions (Figure 2C). For the lncRNA MAGI2-AS3, we found that it significantly downregulated (log2FC = −2, *P* = 1.74E-38) in differential expression analysis. Some studies showed that overexpression of MAGI2-AS3 in BRCA cells MCF-7 would inhibit the Wnt/β-catenin pathway and inhibit cell proliferation and migration. MAGI2-AS3 may act as a cis-acting regulatory element downregulating the DNA methylation level of the MAGI2 promoter region (Du et al., 2019; Xu et al., 2021). Meng et al. (2020) verified that overexpression of MIR99AHG promoted gastric cancer cell proliferation and invasion *via* the miR577/FOXP1 axis. Other experiments have shown that MiR-577 inhibits EMT and metastasis of BRCA by targeting RAB25 (Yin et al., 2018). From these results, it suggested that MIR99AHG may have an effect on BRCA *via* the miR577/RAB25 axis, which provided a suggestion for further experiments. For LINC00641, Mao et al. (2020) confirmed that LINC00641 inhibits BRCA cell proliferation, migration, and invasion by sponging miR-194-5p.

**TABLE 1:** The top 20 lncRNAs/PCGs in degree, betweenness, and closeness.

Symbol	Degree	Symbol	Betweenness	Symbol	Closeness
*MAGI2-AS3	595	*LINC00641	0.2442	CACHD1	0.2876
PCAT19	495	AC009133.1	0.1829	TMEM220	0.2868
LINC01140	472	HIST1H4E	0.1679	CRIM1	0.2867
*MIR99AHG	464	CARMN	0.1328	DMD	0.2865
AC108134.3	448	HIST2H2AC	0.1296	CEP68	0.2864
EMX2OS	443	TYMSOS	0.0931	STAT5B	0.2863
LINC00667	421	MIR4435-2HG	0.0894	ADAMTS5	0.2863
MIR22HG	408	NUP210	0.0848	RBMS2	0.2862
WDFY3-AS2	396	RHPN1-AS1	0.0737	TGFBR3	0.2862
TRHDE-AS1	394	HIST1H4D	0.0698	PLAGL1	0.2860
AC096921.2	383	*MAGI2-AS3	0.0595	LIFR	0.2857
LINC01697	373	AC010326.3	0.0526	CDC14B	0.2856
AC022007.1	368	FAM89A	0.0519	C20orf194	0.2855
LINC02202	362	AC008771.1	0.0456	*LINC00641	0.2855
AC093278.2	341	AC006329.1	0.0448	ANKRD29	0.2853
LINC01537	337	*MIR99AHG	0.0439	TTC28	0.2851
MIR100HG	328	AC105219.4	0.0437	NR3C2	0.2849
A2M-AS1	319	AC092718.4	0.0410	RUNX1T1	0.2846
HCG11	306	ARHGAP5-AS1	0.0352	PTPN14	0.2844
MIR497HG	283	AC010503.4	0.0340	EZH1	0.2843

*Asterisk indicates that lncRNAs appeared in two dimensions. The lncRNA with a higher degree, betweenness, and closeness played crucial roles in the SLGCeNBC.*

To further explore the function of the above three lncRNAs, we performed Gene Ontology (GO) enrichment analysis for the lncRNAs through their related PCGs that shares at least two of three lncRNAs in SLGCeNBC. The result of the GO biological process contained “positive regulation of endothelial cell proliferation,” “cell adhesion,” “angiogenesis,” “regulation of small GTPase mediated signal transduction,” “positive regulation of endothelial cell migration,” “axon guidance,” “phosphatidylinositol phosphorylation,” “positive regulation of apoptotic process,” “regulation of phosphatidylinositol 3-kinase (PI3K) signaling,” and “steroid hormone mediated signaling pathway” (Figure 2D). Many researches had shown that these biological processes were closely associated with BRCA. For example, some deregulation Arf isoforms from the small GTPase subfamily induce cancer formation and progression by enhancing cell proliferation through the activation of mitogen-activated protein kinases (MAPK) and ribosomal protein S6 kinase beta-1 (p70S6K) (Davis et al., 2016; Li et al., 2017). High-level amplification of ARF1 from the Arf subfamily is associated with increased PCG expression and poor outcomes of patients with BRCA (Xie et al., 2016). Overexpression of Ras from the small GTPase subfamily has been found in more than 15% of human tumors (Goitre et al., 2014). The study indicated that Ras which is upregulated in BRCA can promote BRCA cell proliferation, migration, and invasion due to their capability to alter integrin-mediated cell adhesion (Di et al., 2015). For the biological processes, PI3K is the most common altered pathway in ER-positive BRCA and PI3K/AKT is one of the most critical signal pathways for cancer (Hamilton and Infante, 2016; Devanathan et al., 2020). These results recommended that the three lncRNAs with higher degree, betweenness, and closeness were important in the network and played a crucial role in the origin and development of BRCA.

### Identifying Risk lncRNAs by Random Walk With Restart

We mapped 318 PCGs associated with BRCA from DisGeNET (v7.0) (Piñero et al., 2020) into SLGCeNBC. The result showed that there are 40 PCGs mapped into SLGCeNBC. The 40 PCGs acted as the seed nodes (Supplementary Tables 1, 3), and the method of RWR (see section “Materials and Methods”) was performed to prioritize BRCA highly related lncRNAs. The initial score of the seed nodes was set at 1, and the scores of all lncRNA node were calculated. To establish whether the lncRNA scores were significantly higher than the random case, we perturbed SLGCeNBC and performed the RWR 10,000 times. As a result, we identified 30 lncRNAs whose scores were significantly higher than those of the random case (*P* < 0.05, Table 2 and Supplementary Table 4). Here, because the significant results were produced by inputting known BRCA genes and 10,000 times network permutations, these genes were located in the neighbors of the disease genes and considered as the potential synergetic regulators of the disease genes. Thus, all 30 lncRNAs were considered to be risk lncRNAs of BRCA. We showed that the real scores for risk lncRNAs from RWR were higher than the scores for the non-risk lncRNAs (*P* = 2.266e-06, Wilcoxon rank-sum test). Thirteen of 30 risk lncRNAs have been reported to be associated with the occurrence, progression, and survival of tumor. Particularly, some studies had shown that the nine risk lncRNAs were closely related to the proliferation, metastasis, and survival of BRCA (Table 2). For instance, Zhang et al. (2017) revealed that AP000439.3 could regulate the expression of CCND1 through enhancing estrogen receptor induction of CCND1 and function as a key regulator of the cell cycle in BRCA. Ji et al. (2020) demonstrated that LINC00665 promoted BRCA progression and induced an epithelial–mesenchymal transition-like phenotype *via* the upregulation of LIN28B expression (Ding et al., 2020).

**TABLE 2:** Risk lncRNA information.

LncRNA	*P*-value	log2FC	Evidence (PMID)	LncRNA	*P*-value	log2FC	Evidence (PMID)
TYMSOS	0	1.94		AC008115.3	0.0232	1.45	
AC092718.4	0	1.37		AC103760.1	0.0246	1.19	
AC006329.1	0	1.25	30195788	*LINC00665	0.0276	1.14	31907362, 32083756
AC108860.2	0	1.28		ELF3-AS1	0.03	1.17	32598181, 32194747
*AP000439.2	0.0002	2.97	29048636, 30582215	*AC093297.1	0.0332	4.44	32733537
AC099850.3	0.0004	2.80	33102579, 31391008	AP005121.1	0.0338	2.38	
AL161908.1	0.0028	1.15		*SIAH2-AS1	0.0356	1.52	31572452
MAFG-DT	0.0046	1.35	32382761	*MRPS30-DT	0.0384	2.40	31788446
AC009133.1	0.0058	1.06		*AC093297.2	0.0396	1.26	32733537
AL031985.3	0.0066	1.19		AC008771.1	0.0404	1.05	
ATP2A1-AS1	0.0068	1.88		AC037198.1	0.0444	2.02	
AC141930.1	0.0076	1.46		AP005131.7	0.0444	2.15	
*AP000851.1	0.0096	−1.54	31608996, 32867770	LINC01117	0.0444	2.06	
*AFAP1-AS1	0.0168	3.81	32020881, 32955920	LINC01977	0.0446	1.67	
AL390294.1	0.02	1.54		*AC144450.1	0.0484	1.52	32420379

*Asterisk indicates that there is literature supporting an association between lncRNA and BRCA. The ***P***-value indicates statistical significance by comparing the scores of lncRNAs in the network following n iterations with SLGCeNBC perturbation (see section “Materials and Methods”).*

**FIGURE 3:**
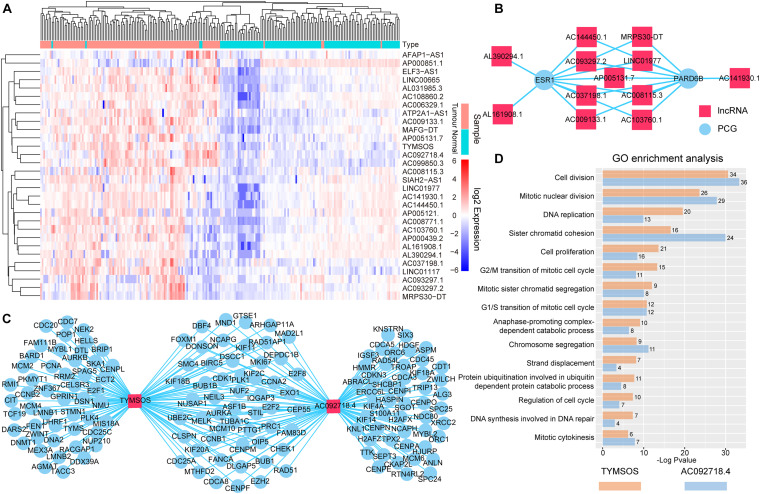
Analysis of the identified risk lncRNAs. **(A)** The heatmap of 30 risk lncRNAs based on their expression. The columns represented 224 samples of 112 BRCA patients, and the rows represented lncRNAs. The risk lncRNAs classified the samples into tumor tissue and adjacent nontumor tissue. **(B)** Top two PCGs with high lncRNA interactive relationships. Red nodes represent lncRNAs, and blue nodes represent PCGs. **(C)** Top two risk lncRNAs with high PCG interactive relationships. **(D)** GO enrichment analysis for lncRNAs TYMSOS and AC092718.4 through their related PCGs, respectively. The *X*-axis shows the –log10 of the *P*-value; the *Y*-axis shows the names of the GO term. The number at the top of the bar indicates the number of PCGs annotated to the GO term.

We performed bidirectional hierarchical clustering to further investigate the risk lncRNAs. In the heatmap (Figure 3A), we found that the lncRNAs classified the samples into adjacent nontumor tissue and tumor tissue, suggesting that these lncRNAs possessed potential for diagnosis and therapy of BRCA. Thereafter, we counted the amount of risk lncRNAs that interacted with each PCG. We found that the top two PCGs were ESR1 and PARD6B, which connected with 11 and 10 risk lncRNAs, respectively (Figure 3B). The evidence indicated estrogen receptor-alpha (ERalpha) binding to all identified SRC-3 genomic binding sites from E2-treated cells and confirmed the ability of SRC family coactivators to regulate the expression of one of these PCGs, PARD6B/Par6 (Labhart et al., 2005). We also counted the number of the PCGs that interacted with risk lncRNAs. We found that the top two risk lncRNAs were TYMSOS and AC092718.4, each of which connected with 103 PCGs (Figure 3C). We performed GO enrichment analysis and Kyoto Encyclopedia of Genes and Genomes (KEGG) pathway enrichment analysis for these PCGs related to two risk lncRNAs, respectively. We found that TYMSOS and AC092718.4 were mainly enriched in the biological processes and pathways related to the cell cycle, such as “cell division” and “proliferation.” Interestingly, the result of TYMSOS was similar as that of AC092718.4 (Figure 3D and Supplementary Table 5, Jaccard similarity coefficient = 0.52). However, the similarity of PCGs which was regulated by TYMSOS and AC092718.4 was significantly lower than that of enrichment results (Figure 3C, Jaccard similarity coefficient = 0.35). These results suggest that risk lncRNAs may regulate in coordination the occurrence and development of BRCA.

### Synergistic Regulation of Risk lncRNAs

**FIGURE 4:**
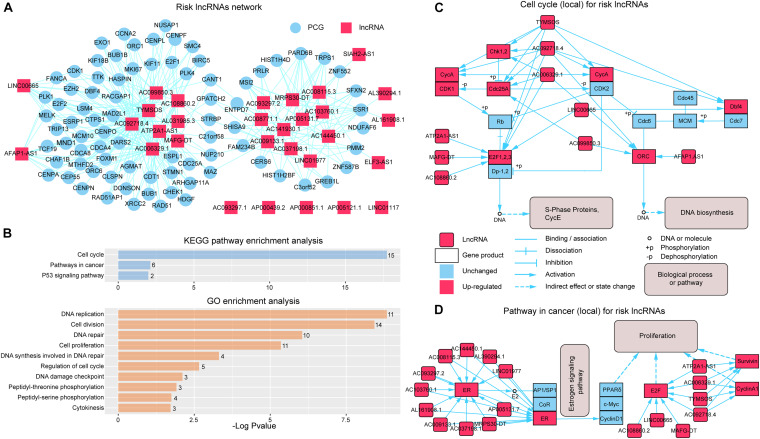
Synergistic regulation of the risk lncRNAs. **(A)** Risk lncRNA network; PCGs regulated by more than three risk lncRNAs were selected. The red and blue nodes represent lncRNAs and PCGs, respectively. **(B)** KEGG and GO enrichment analysis was performed using genes in the risk lncRNA network. The *X*-axis shows the –log10 of the *P*-value; the *Y*-axis shows the names of the pathway/term. The number at the top of the bar indicates the number of PCGs annotated to the pathway/term. **(C)** Risk lncRNAs regulate the cell cycle pathway (local). **(D)** Risk lncRNAs regulate the pathway in cancer (local).

To further investigate the synergistic regulation function for risk lncRNAs, we focused on the PCGs regulated by more than three risk lncRNAs (Figure 4A) and performed GO function and KEGG pathway enrichment analysis. The results showed that the PCGs were significantly enriched to the KEGG pathway containing “Cell cycle,” “Pathways in cancer,” and “p53 signaling pathway” (Figure 4B). In more details, eight risk lncRNAs (TYMSOS, ATP2A1-AS1, AC092718.4, MAFG-DT, AC108860.2, AC006329.1, LINC00665, AC099850.3, and AFAP1.AS1) from the “Cell cycle” pathway (Figure 4C) showed a ceRNA relationship with E2F1, 2, 3 (E2F1 and E2F2), Chk1 (CHEK1), Cdc25A (CDC25A), CycA (CCNA2), ORC (ORC1 and ORC2), and Dbf4 (DBF4) in SLGCeNBC, respectively. On the other hand, TYMSOS, AC092718.4, and AC006329.1 jointly upregulated the expression of CHEK1 (Chk1) and CCNA2 (CycA), inhibited the phosphorylation of Cdc25A (CDC25A), to reduce the dephosphorylation of CDK1 and CDK2, and further improved the phosphorylation levels of Rb and Dp-1,2. Consistently, ATP2A1-AS1, MAFG-DT, AC108860.2, and LINC00665 upregulated the expression of E2F1 and E2F2 (E2F1, 2, 3). To sum up, the expression of S-phase proteins CycE was indirectly co-promoted. On the other hand, the expression of DBF4 (Dbf4) was upregulated by TYMSOS, AC092718.4, and LINC00665, which accelerated the phosphorylation of MEM, and the ORC (origin recognition complex) was upregulated by AC092718.4, AC006329.1, AC099850.3, and AFAP1.AS1, which ultimately promoted DNA biosynthesis. For the “Pathway in cancer” pathway (Figure 4D), a total of 18 risk lncRNAs synergistically regulated ER (ESR1), E2F (E2F1 and E2F2), CyclinA1 (CCND1), and Survivin (BIRC5), which indirectly leads to tumor cell proliferation. In the eight risk lncRNAs, there are clear reports that LINC00665 is related to BRCA. LINC00665, which acted as ceRNA, promoted BRCA progression and induced an epithelial–mesenchymal transition-like phenotype *via* the competitively upregulation of LIN28B expression (Ding et al., 2020; Ji et al., 2020).

By GO function analysis, we found that many biological processes were significant including “DNA replication,” “cell division,” “DNA repair,” “cell proliferation,” “DNA synthesis involved in DNA repair,” “regulation of cell cycle,” “DNA damage checkpoint,” “peptidyl-threonine phosphorylation,” “peptidyl-serine phosphorylation,” and “cytokinesis” (Figure 4B). Obviously, these biological processes are closely related to tumor cell proliferation differentiation and apoptosis. For the GO terms “peptidyl-threonine phosphorylation” and “peptidyl-serine phosphorylation,” Saeidi et al. (2020) have found that peptidyl-prolyl cis-trans isomerase NIMA-interacting 1 is highly overexpressed in human breast tumor tissues and H-Ras transformed human mammary epithelial (H-Ras MCF10A) and MDA-MB-231 BRCA cells.

### Construction and Evaluation of Risk Prediction Model in the Training Dataset

**FIGURE 5:**
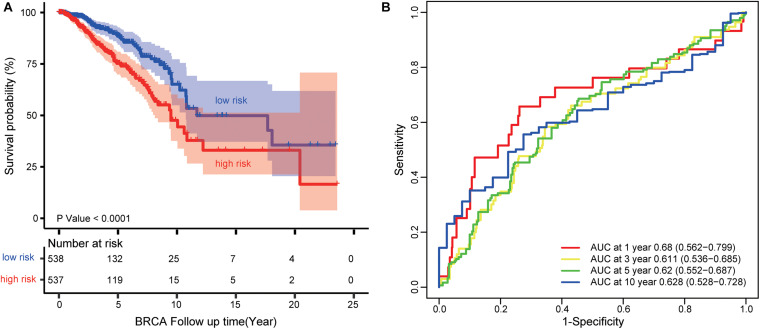
Survival prediction performance of the four-lncRNA signature (AP000851.1, LINC01977, MAFG-DT, and SIAH2-AS1). **(A)** Kaplan–Meier curves for BRCA patients stratified by the four-lncRNA signature. *P*-values were calculated using a log-rank test. **(B)** ROC analysis to assess the survival accuracy of the four-lncRNA signature for 1, 3, 5, and 10 years of survival.

We used the TCGA dataset to develop the risk prediction model and construct the prognostic signature, since we discovered 17 lncRNAs out of the 30 selected ones associated with the survival of BRCA patients. Then, we used the 17 prognostic lncRNAs to develop the risk prediction model and obtained 2^17^-1 = 131,071 risk models. We performed Kaplan–Meier analysis and compared the predictive ability of 131,071 signatures. A four-lncRNA signature (AP000851.1, LINC01977, MAFG-DT, and SIAH2-AS1) was found to have the minimum log-rank *P*-value (Figure 5A). The regression coefficients of the four lncRNAs (AP000851.1, LINC01977, MAFG-DT, and SIAH2-AS1) were all negative, which means they were related to BRCA poor prognosis (Supplementary Table 6).

### Survival Prediction Performance of the Four-lncRNA Signature in the TCGA Dataset

Each patient in the TCGA dataset received a risk score based on the four-lncRNA signature. Then, the patients with BRCA in the TCGA dataset were divided into high-risk (*n* = 537) or low-risk group (*n* = 538) based on the median risk score. Kaplan–Meier analysis demonstrated that patients in the low-risk group owned longer survival times than those in the high-risk group (median survival time: 11.69 vs. 9.48 years, log-rank test *P* < 0.001; Figure 5A). Subsequently, we performed time-dependent receiver operating characteristic (ROC) analysis to assess the survival accuracy of the four-lncRNA signature. In the TCGA dataset, the area under the ROC curve (AUC) for 1, 3, 5, and 10 years of survival were 0.68, 0.61 0.62, and 0.63, respectively (Figure 5B).

To test its prognostic independence, univariate and multivariable Cox regression analyses (Cox, 1972) were conducted. The multivariable Cox regression results in the TCGA datasets verified that the four-lncRNA signature can predict patients’ survival [high- *vs.* low-risk, hazard ratio (HR) training = 2.02, 95% confidence interval (CI) 1.43-2.86, *P* < 0.001, *n* = 1075; Table 3].

**TABLE 3:** Cox regression analysis of the signature with BRCA survival (*n* = 1075).

		Univariable analysis				Multivariable analysis			
Variables		HR	95% CI of HR		*P*	HR	95% CI of HR		*P*
			Lower	Upper			Lower	Upper	
Age	>58 vs. ≤58	1.75	1.27	2.41	<0.001	2.03	1.44	2.85	<0.001
Stage	I, II vs. III, IV	2.17	1.74	2.72	<0.001	2.11	1.69	2.63	<0.001
lncRNA signature	High risk vs. low risk	1.98	1.43	2.76	<0.001	2.02	1.43	2.86	<0.001

*HR, hazard ratio; CI, confidence interval.*

## Discussion

In recent years, the role of lncRNA has become a highly studied topic in the field of tumor research. Accumulating evidence indicates that lncRNA is involved in the oncogenesis and development of BRCA (Bray et al., 2018; DeSantis et al., 2019; Ji et al., 2020). With the development of high-throughput sequencing technology, a large number of lncRNA expression data involved in the occurrence and progression of cancer are emerging. It is not hard to infer that many of the lncRNAs related to BRCA may function as a complex and organized regulatory network for BRCA (Chi et al., 2019).

In this study, SLGCeNBC was constructed based on co-expression and miRNA-mediated RNA cross talks. Therefore, we identified 30 risk lncRNAs associated with BRCA by performing RWR in SLGCeNBC. In 30 risk lncRNAs, 13 risk lncRNAs have been confirmed to be associated with several cancers and nine risk lncRNAs have demonstrated a high association with BRCA. This shows that our method is effective and practical. By means of enrichment analysis using GO and KEGG, we found that these risk lncRNAs are significantly enriched in cancer-related biological processes and pathways, which have been stated by researchers (Hamilton and Infante, 2016; Anwar et al., 2018; Devanathan et al., 2020; Saeidi et al., 2020), and we found that the regulation of risk lncRNAs on BRCA-related genes is synergistic rather than alone. As a result, we found that these 30 risk lncRNAs are highly correlated with the occurrence and development of BRCA, and they may form the network system to jointly regulate the initiation and course of BRCA. These results suggest that risk lncRNAs may serve as novel diagnostic markers and treatment targets.

Importantly, we identified a four-lncRNA prognosis signature based on ceRNA network analysis, which could be used as the key clinical biomarker in BRCA prognosis. The poor prognosis of BRCA is mainly manifested in tumor metastasis, which is the leading cause of death (Zhang et al., 2017). Tumor metastasis is difficult to detect, which is discovered only when the tumor is large enough to be observed in regular follow-up imaging examination or to cause notable symptoms resulting from a tumor mass effect. This situation may exacerbate the patient prognosis. In our study, after survival analysis, we found that a four-lncRNA signature including four lncRNAs (AP000851.1, LINC01977, MAFG-DT, and SIAH2-AS1) has the ability to predict survival in patients with BRCA, which are expected to be novel predictors that may identify early metastasis. Additionally, we also investigate prognostic independence effects of the four-lncRNA signature in the TCGA dataset. As a result, the multivariable Cox regression results in the TCGA datasets verified that the four-lncRNA signature can predict patients’ survival. This result also demonstrated the clinical potential of the four-lncRNA signature.

Our methods also show some limitations. First of all, in this study, we conducted a bioinformatics analysis to identify the crucial factors in BRCA; results indicated that some genes (PCGs or lncRNAs) might play vital roles in the subtype cancers. Bioinformatics may infer only the functions of these lncRNAs; thus, it remains necessary to confirm the biological effects of these risk lncRNAs in BRCA in experimental studies. This result also encouraged us to validate the biological function and mechanism. In a further study, we will conduct the biological experiments to investigate these potential factors. Secondly, the risk lncRNAs identified here may not be all candidate risk lncRNAs associated with BRCA because of only limitations of lncRNA data. Finally, this study is based on the DE lncRNAs in BRCA. If all lncRNAs in tumors participate in the occurrence and development of tumors in the form of a network system, then some lncRNAs with no obvious changes may slip through the net and our results range may be narrowed. However, the research results of our study could contribute to accelerating the discovery of molecular biomarkers for diagnosis, treatment, and prognosis evaluation of BRCA.

## Data Availability Statement

Publicly available datasets were analyzed in this study. This data can be found here: https://xenabrowser.net/datapages/.

## Author Contributions

XS and LS contributed manuscript. LS and LW revised the manuscript. All authors contributed to the manuscript revision and, read and approved the submitted version.

## Conflict of Interest

The authors declare that the research was conducted in the absence of any commercial or financial relationships that could be construed as a potential conflict of interest.

## Footnotes

1 https://genome-cancer.ucsc.edu

2 www.r-project.org

## Abbreviations

BRCA: breast cancer
lncRNA: long non-coding RNA
PCG: protein coding gene
ceRNA: competitive endogenous RNA
TCGA: The Cancer Genome Atlas
DE: differentially expressed
PCC: Pearson correlation coefficient
RWR: random walk with restart
SLGCeNBC: specific LncRNAs-PCGs ceRNA network for breast cancer
ROC: receiver operating characteristic
KM: Kaplan–Meier
AUC: area under the ROC curve
GO: Gene Ontology
KEGG: Kyoto Encyclopedia of Genes and Genomes
HR: hazard ratio
CI: confidence interval.
